# High Osmolarity Environments Activate the Mitochondrial Alternative Oxidase in *Debaryomyces Hansenii*

**DOI:** 10.1371/journal.pone.0169621

**Published:** 2017-01-06

**Authors:** Wilson Garcia-Neto, Alfredo Cabrera-Orefice, Salvador Uribe-Carvajal, Alicia J. Kowaltowski, Luis Alberto Luévano-Martínez

**Affiliations:** 1 Departamento de Bioquímica, Instituto de Química, Universidade de São Paulo, São Paulo, Brazil; 2 Departamento de Genética Molecular, Instituto de Fisiología Celular, Universidad Nacional Autónoma de México, México, México; National Research Council, ITALY

## Abstract

The oleaginous yeast *Debaryomyces hansenii* is a good model to understand molecular mechanisms involved in halotolerance because of its impressive ability to survive under a wide range of salt concentrations. Several cellular adaptations are implicated in this response, including the presence of a cyanide-insensitive ubiquinol oxidase (Aox). This protein, which is present in several taxonomical orders, has been related to different stress responses. However, little is known about its role in mitochondria during transitions from low to high saline environments. In this report, we analyze the effects of Aox in shifts from low to high salt concentrations in the culture media. At early stages of a salt insult, we observed that this protein prevents the overflow of electrons on the mitochondrial respiratory chain, thus, decreasing the production of reactive oxygen species. Interestingly, in the presence of high osmolite concentrations, Aox activity is able to sustain a stable membrane potential when coupled to complex I, despite a compromised cytochrome pathway. Taken together, our results suggest that under high osmolarity conditions Aox plays a critical role regulating mitochondrial physiology.

## Introduction

Halophilic organisms are genetically adapted to hypersaline conditions and require these conditions for optimal growth, while halotolerant organisms are only able to grow under these conditions due to adaptive mechanisms triggered by sudden changes in osmolarity [1]. Proteins and membranes are key players in the halotolerant response in several organisms. In general, halophile proteins present a higher anionic aminoacid content than their non-halophilic counterparts. It is suggested that this feature may be the evolutionary trait that permitted these organisms to colonize saline environments. On the other hand, halotolerant proteins do not seem to present special features responsible for osmotic stress resistance in these organisms [2, 3].

Mitochondria are directly involved in metabolic responses to osmotic stress. In *Saccharomyces cerevisiae*, defects in electron transport chain activity make cells unable to respond to osmotic shock [4]. Similarly, a yeast mutant of the MAP kinase Hog1 presents mitochondrial phenotypes, which indicates that mitochondria and the transcriptional regulation of target genes of the general stress response are interconnected [5]. The physiological significance of this relationship is not fully understood but apparently is not only due to mitochondrial ATP production. The production of metabolites necessary for the synthesis of osmolites such as glycerol, trehalose, glycine betaine or proline is also related [6]. These substances are critical to protect cellular structures from environmental stressors. The mechanism involved in biomolecule protection by osmolites remains unknown so far, but perhaps they alter water molecule organization, generating a glassy structure around the biomolecule and thus preserving the functionality of the biomolecule [7].

**Fig 1:**
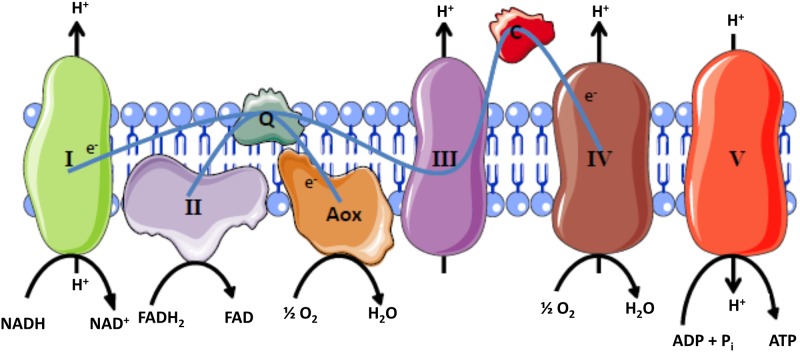
Simplified representation of the respiratory chain of *Debaryomyces hansenii*. *Debaryomyces hansenii* has a proton-pumping complex I, a canonical proton-pumping cytochrome pathway (complex III-IV) and a cyanide-resistant terminal oxidase (Aox). For simplicity, the alternative NADH dehydrogenase and glycerol-3-phosphate dehydrogenase described in [9] are not depicted. Electrons flow from complex I to ubiquinone and at this point the respiratory chain is branched and electrons flow to complex III and the Aox. Thus, two electron pathways are able to sustain a protonmotive force: Complex I-Aox and Complex I-III-IV.

*Debaryomyces hansenii* is an oleaginous yeast present in a wide variety of ecological niches, such as seawater, capable of vast metabolic adaptations and therefore a good model to study halotolerance [8]. Differently from *Saccharomyces cerevisiae*, this yeast presents several mitochondrial features that could be involved in its adaptive mechanisms to high salinity. Its respiratory chain presents a functional proton-pumping complex I (NADH: ubiquinone oxidoreductase) and a cyanide-insensitive and non-proton pumping terminal oxidase termed alternative oxidase (Aox), which uses ubiquinol and oxygen as substrates [9] (Fig 1).

The aim of this work is to elucidate the role of mitochondria in the adaptive response to high salinity. We observed that *Debaryomyces hansenii* improves its growth rate when sodium, potassium or sorbitol were present in the culture media. Interestingly, we find that the Aox in the respiratory chain of this organism is overexpressed under the hyperosmotic response suggesting a role of this protein in mitochondrial homeostasis.

## Materials and Methods

### Strain and growth conditions

The *Debaryomyces hansenii* Y7426 strain, from the USA Department of Agriculture (Peoria, IL), was used throughout this study. Cells were pre-cultured in YPD (1% yeast extract, 2% peptone, 2% glucose) until saturation. Cultures were diluted to an optical density of 0.01 in YPD media and incubated at 28°C under vigorous shaking at 180 rpm. For hypersaline cultures, sodium chloride or potassium chloride were added to a final concentration of 0.6 M. Sorbitol was added in some cultures at a final concentration of 1.2 M. Growth was continuously monitored by measuring absorbance changes at 600 nm.

### Survival assay

Survival over time (chronological lifespan) was assessed by measuring colony-forming units over time. Stationary phase samples (2 mL) from cultures were centrifuged, suspended in MiliQ water and serially diluted until a final optical density of 0.0001. A 100 μL-aliquot of the last dilution was taken and plated on solid YPD media. Colonies were incubated at 28°C for 3 days and colony-forming units (C.F.U) were counted manually. Additionally, the same dilution was layered on solid media supplemented with 3 mM salicylhydroxamic acid (SHAM) or 10 mM hydrogen peroxide and incubated at 28°C for 3 days and viability was determined by manually counting C.F.U. 5 μL-serial dilutions were spotted on solid media plates supplemented with 3 mM SHAM and incubated for 3 days.

### Cell extracts

Cell extracts were prepared from stationary phase cultures. Cells were suspended in grinding buffer (0.1 M Tris-HCl, pH 8.0 supplemented with Roche protease inhibitor cocktail) and broken with glass beads for 1 h at 4°C under vigorous shaking. Extracts were centrifuged at 4000 x*g* for 10 min to eliminate cell debris. Protein was quantified by the Bradford method.

### Mitochondrial isolation

Mitochondria were isolated after enzymatic treatment of cultures grown on YPD under the conditions stated previously. Briefly, cells were centrifuged, washed with MiliQ water and suspended at a final concentration of 25 mg_cell_/mL in 1.2 M sorbitol, 50 mM EDTA and 10 mM 2-mercaptoethanol, pH 7.4 containing 3 mg/g_cell_ zymolyase. Cells were incubated at 30°C and 180 rpm for 60 min. Spheroplasts obtained were centrifuged at 3200 x*g* at 4°C for 5 min. Pellets were washed and suspended in lysis buffer (0.6 M mannitol, 20 mM Hepes, 2% bovine serum albumin (BSA), pH 6.8). Spheroplast formation was followed spectrophotometrically at 600 nm. Afterward, spheroplasts were homogenized with a Dounce homogenizer (25 strokes) and centrifuged at 2500 xg for 5 min to eliminate unbroken spheroplasts and cell debris. The supernatant was centrifuged at 10500 xg for 10 min; the resulting supernatant was discarded and the mitochondrial pellet suspended in isolation buffer and centrifuged at 5500 xg for 5 min. The supernatant was recovered and centrifuged at 12000 xg for 10 min and the pellet (mitochondria) suspended in minimal isolation buffer. Respiratory control ratios (RCR) of isolated mitochondria were routinely measured to verify mitochondria integrity. A RCR of 4–5 using pyruvate/malate/citrate was usually obtained using this isolation protocol. Alternatively, mitochondria were isolated after cell lysis with glass beads in a bead beater at a constant rheostat level as in [10], with similar results in quality.

### Western blotting

50 μg of protein from cell extracts were separated by SDS-PAGE and transferred to PVDF membranes by standard western blot procedures. Membranes were incubated with antibodies raised against plant Aox (Agrisera) and cytochrome *c* oxidase subunit III (CoxIII, Abcam) at 1:200 and 1:1000 dilutions, respectively. Secondary antibodies coupled to fluorescent probes (Lycor) were used at a final dilution of 1:15000. Band intensities were scanned in an Odyssey fluorimeter.

### Oxygen consumption

Cell cultures were centrifuged and suspended in 20 mM Hepes, pH 6.8 at a final concentration of 1 mg·mL^-1^ (wet weight). Respiratory rates were measured using an Oroboros (Innsbruck, Austria) high-resolution oxygraph using 25 mM glucose as respiratory substrate. Where indicated, 0.5 mM KCN, 3 mM SHAM or 0.5 μM antimycin A was added to the incubation chamber. For measurements of short-term effects of osmolarity on mitochondrial respiration, cells were grown in YPD as above, washed in 20 mM Hepes pH 6.8, suspended at a final concentration of 10 mg·mL^-1^ in YPD media with or without 0.6 M NaCl, 0.6 M KCl or 1.2 M sorbitol and oxygen consumption was quantified as above.

For respiration in isolated mitochondria, samples were suspended to a final concentration of 0.5 mg·mL^-1^ in respiration buffer (0.8 M sorbitol, 20 mM Hepes pH 6.8, 20 mM KCl, 10 mM Tris-phosphate). 10 mM succinate/rotenone (1μM) or 10 mM pyruvate/malate/citrate were used as respiratory substrates. 100 μM KCN and 100 μM SHAM were included in some experiments.

### Mitochondrial membrane potential

Mitochondrial membrane potentials were estimated *in situ* in a cell suspension (1 mg.mL^-1^) by monitoring fluorescence changes of 3,3’-dihexiloxacarbocianine (1 nM, DiOC_6_), (λ_ex_ 482 nm, λ_em_ 524 nm) [11] in a Hitachi F4500 fluorescence spectrophotometer. The incubation media was the same as in oxygen consumption experiments. Data are expressed as the difference between the membrane potential in the presence of 3 mM SHAM and the potential in the presence of 0.5 mM antimycin A.

In addition, the membrane potential in isolated mitochondria was also determined as in [10] using 10 mM pyruvate/malate/citrate as substrates. Where indicated, 100 μM KCN, 1 μM propyl gallate, 100 μM SHAM or 10 μM carbonyl cyanide m-chlorophenyl hydrazone (CCCP) was added to the incubation media.

### Hydrogen peroxide production

Mitochondria were diluted to 0.5 mg.mL^-1^ in assay buffer (0.6 mannitol, 20 mM Hepes, 20 mM K_2_HPO_4_, 2 mM MgCl_2_, 50 μM Amplex Red, 0.5 U. mL^-1^ horseradish peroxidase (HRP), pH 7.0). Where indicated, 1 mM NADH, 1 μM antimycin A, 100 μM SHAM and/or 2 μM CCCP were included in the incubation media. Resorufin fluorescence was monitored using a Hitachi F4500 fluorescence spectrophotometer using λ_ex_ 563 nm, λ_em_ 587 nm.

### Trehalose quantification

Trehalose was quantified after trehalose hydrolysis by trehalase treatment of the cell extracts. Briefly, cell extracts were suspended in 5 mM Tris-HCl, pH 7.5 and treated with 100 mU porcine trehalase. After 20 min incubation, 0.4 U.mL^-1^ HRP, 100 mU glucose oxidase and 0.1% *o*–dianisidine were added and incubation was continued for additional 30 min at room temperature. Then, absorbance changes were read at 420 nm using a microplate reader (Molecular Devices).

### Glycerol quantification

Glycerol was quantified from cell extracts obtained as above using a commercial kit (InVitro) adapted for microplates. Absorbance was read at 420 nm in a microplate reader (Molecular Devices).

### Cell size measurements

Logarithmic-phase cultures in YPD media were pelleted, washed and filtered. Then, yeast cells were suspended in 20 mM Hepes pH 7 or in the same buffer but supplemented with 0.6 M NaCl. Cells were sorted in a BD-FACS Verse and light scattering from samples was analyzed with FlowJo 7.6 software.

### Sodium uptake measurements

Stationary phase cultures were centrifuged and washed in 20 mM Hepes pH 6.8. Cells were weighed and diluted at a final concentration of 100 mg/mL in the same buffer. 50 μg SBFI-AM (Invitrogen) was dissolved in 100 μL DMSO/1% pluronic acid and added to the cell suspension. Cells were incubated at 28°C for 30 min. Afterward, cells were centrifuged and washed two times in 20 mM Hepes pH 6.8 to eliminate non-internalized SBFI. For sodium uptake assays, cells were diluted to 1 mg/mL in a 3 mL cuvette with washing buffer. SBFI fluorescence was recorded in a F4500 Hitachi Fluorimeter with excitation at 340 and 380 nm and measuring the emission of each wavelength at 505 nm. Data were recorded as the ratio of emission intensities at each excitation wavelength (I_340_/I_380_) and calibrated *in situ* using a mixture of gramicidin/alamethicin (10 μM each) and successive 2 mM additions of sodium chloride. Cells were energized with 25 mM glucose to allow sodium uptake. Where indicated a single addition of NaCl (Final concentration 0.6 M) or the gramicidin/alamethicin mixture was included.

### Statistical analysis

Three independent experiments were performed and each replica determined in triplicate. Data were analyzed by two-way ANOVA using Origin 7.0. *p* < 0.05 was considered statistically significant. Data represent mean value ± SEM of at least 4 independent experiments.

## Results

### Respiratory activity is increased in hyperosmotic conditions

**Fig 2:**
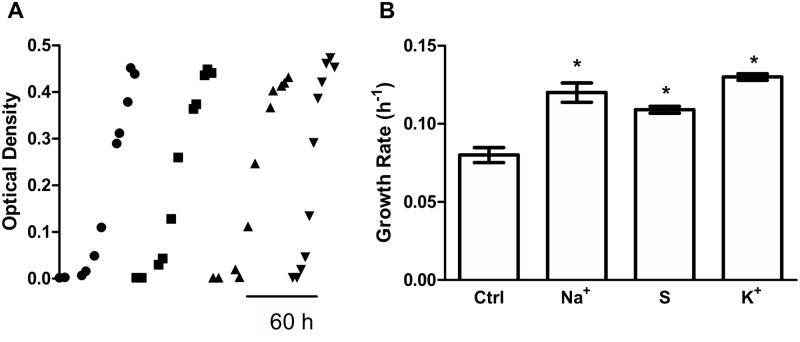
High osmolarity positively affects growth independently of the carbon source. (A) Growth kinetics of cultures in glucose media (YPD, filled circles) or YPD supplemented with 0.6 M sodium chloride (filled squares), 1.2 M sorbitol (filled triangles) or 0.6 M KCl (filled inverted triangles). (B). Growth rate calculated from Panel A. * *p* < 0.05 respect to cultures in YPD media.

To understand the physiological adaptations that *Debaryomyces hansenii* undergoes to survive in a wide range of osmolarities, cells were grown on glucose media with or without sodium, potassium or sorbitol as osmolites. Under our experimental conditions, the growth rate of these cells increases independently of the osmolite present in the media (Fig 2, typical traces are shown in Panels A, quantifications are depicted in Panel B). This result agrees with previous reports [12, 13].

**Fig 3:**
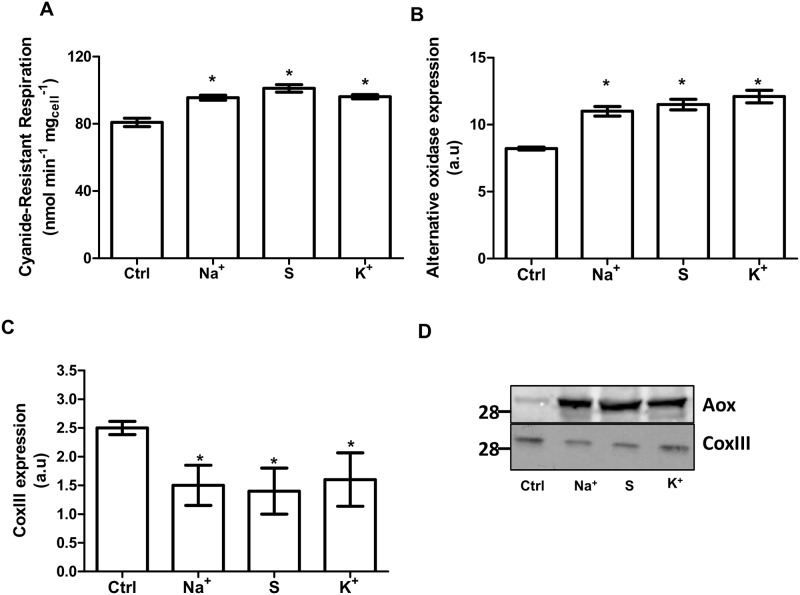
Increases in osmolarity in glucose media promote cyanide-resistance respiration. (A) Cyanide-resistant respiration in cells grown in YPD. Cells (1 mg.mL^-1^) were incubated in respiration buffer. To induced Aox-dependent respiration, 25 mM glucose and 0.5 mM potassium cyanide (KCN) were included in the incubation media. (B-D) Densitometry analysis of the expression of the Aox (Panel B) and cytochrome *c* oxidase subunit III (CoxIII, Panel C) in glucose media in the presence of 0.6 M NaCl, 1.2 M sorbitol or 0.6 M KCl. (D) Representative blots of Aox and CoxIII expression.

In the presence of salts, *D*. *hansenii* activates the glyoxylate cycle, some Krebs cycle enzymes, and increases respiratory activity [14]. Differently from *Saccharomyces cerevisiae*, this yeast is Crabtree negative (i.e. it is a poor fermenter) so glucose is expected to be diverted mainly to mitochondria to produce biomass [15]. In order to verify the influence of osmolites on the respiratory status in D. *hansenii*, oxygen consumption was measured in intact cells (Fig 3A). As observed in this figure, all osmolites increased the respiration resistant to cyanide suggesting that the mitochondrial alternative oxidase (Aox) is more active under these growth conditions. To corroborate this hypothesis, the expression levels of Aox and cytochrome *c* oxidase subunit III (CoxIII) were determined. In agreement with the activity measurements, we found that Aox level increased under this growing conditions (Fig 3B). In this regard, the higher expression of Aox and decreased levels of CoxIII (Fig 3C) in glucose media support the increased basal respiration present in these samples. However, this does not explain why cells grown in glucose/osmolite media are still (although poorly) stimulated by the uncoupler, 2,4-dinitrophenol (DNP) (not shown). An explanation for the activating effect of DNP under these conditions is that electrons coming from complex I are channeled not only to the cytochrome pathway (cytochrome *bc*1 + Cox) but also to Aox [16] (see Fig 1). If this is true, protons should be pumped by the functional coupling between Complex I and Aox.

**Fig 4:**
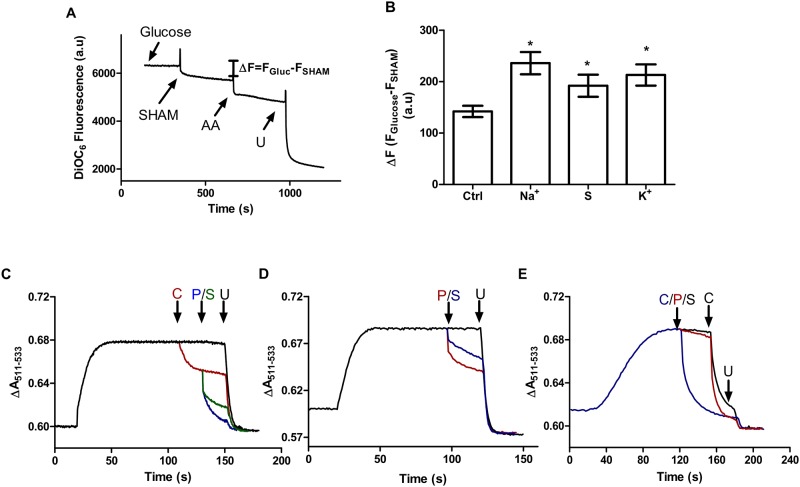
Aox sustains a membrane potential (ΔΨ) when coupled to complex I. (A) Representative plot of mitochondrial membrane potential measurements in intact cells grown in glucose media Where indicated, 25 mM glucose, 0.5 μM antimycin A (AA), 3 mM SHAM and 10 μM CCCP (U) were added. Cells were incubated in regular respiration media at a final concentration of 1 mg.mL^-1^ (B) Aox-dependent ΔΨ measured as the difference between maximum fluorescence (glucose) and the fluorescence after Aox inhibition (SHAM). (C-E) Representative ΔΨ plots of isolated mitochondria from cells grown in YPD. (C) Complex I-dependent ΔΨ (10 mM pyruvate/malate/citrate each). Traces: Black, control without inhibitors. Red, Aox-sustained ΔΨ, in the presence of 100 μM cyanide (addition where indicated). Green/Blue, contribution of the cytochromes to the ΔΨ after the addition of 100μM salicylhydroxamic acid (SHAM, green) and 1 μM propyl gallate (PG, blue). (D) ΔΨ- dependent on Aox. Traces: Black, control without inhibitors. Blue/Red after addition of 100μM SHAM (blue) and 1 mM PG (Green) respectively. (F) Succinate/rotenone (10 mM/ 1 μM) dependent ΔΨ. Traces: Blue, complete collapse of ΔΨ after 100 μM cyanide addition. Black/Red, inhibition of Aox by SHAM (black) or PG (red) did not exert any effect on the ΔΨ, complete collapse of ΔΨ under these conditions was obtained after addition of 100 μM cyanide. At the end of all plots, 1 μM CCCP (U) was added to completely collapse the ΔΨ. * p < 0.05 respect to control cells.

To verify this hypothesis, mitochondrial membrane potentials (ΔΨ) were measured in intact cells under the same experimental conditions as those used in oxygen consumption experiments. Cells were incubated with glucose and the uptake of the ΔΨ-sensitive probe into mitochondria was followed fluorometrically. As observed in Fig 4A and 4B, the addition of a specific inhibitor of the Aox, SHAM, partially decreased ΔΨ at concentrations that totally inhibit Aox activity. The small decrease in control cells suggests that Aox is partially involved in the maintenance of ΔΨ in control cells. Moreover, in cells growing in hyperosmotic media this drop is increased, suggesting that the Aox supports respiratory complexes to maintain a membrane potential. However, the fact that significant probe uptake is insensitive to SHAM plus antimycin indicates that the signal of the probe is significantly altered by non-mitochondrial membrane potentials. Thus, to verify the contribution of the Aox to sustain a mitochondrial ΔΨ, mitochondria were isolated and ΔΨ was analyzed once again using NADH-producing substrates pyruvate/malate/citrate (Complex I) to follow electron flux from Complex I to the cytochrome pathway and Aox. Fig 4C shows that once isolated mitochondria established a ΔΨ, the addition of cyanide produces a partial reduction in ΔΨ (red trace) which is completely collapsed by the Aox inhibitors SHAM (green trace) and propyl gallate (PG, blue trace). When PG or SHAM was added at the beginning of the trace instead of cyanide, a small decrease in ΔΨ was observed. This result is explained by the fact that the inhibition of Aox diverts electrons to cytochrome *bc*1 (Fig 4D). As a control, a third experiment was performed using succinate/rotenone instead of the complex I substrates (Fig 4E). In this case, the addition of PG (red trace) or SHAM (black trace) was unable to collapse the membrane potential. These results ruled out a possible uncoupling effect exerted by these inhibitors (or a possible inhibitory effect on succinate dehydrogenase) and further corroborated that electrons feeding Aox came preferentially from Complex I, sustaining a ΔΨ.

### Osmolites activate the cyanide-resistance respiration (Aox)

**Fig 5:**
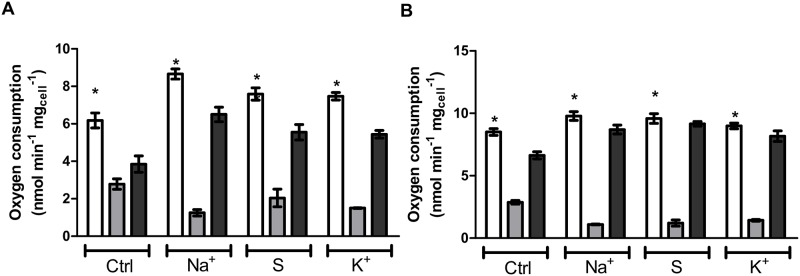
Increased osmolarity decreases the activity of the cytochrome pathways. A. Oxygen consumption in logarithmic phase cells. Cells (1 mg mL^-1^) were grown in YPD media, harvested, washed and suspended in the indicated incubation media. White bars, respiration in YPD media. Light gray bars, cytochrome-dependent respiration in the presence of SHAM. Dark gray bars, Aox-dependent respiration in the presence of cyanide. B. Oxygen consumption in cells in the logarithmic phase. Cells (1 mg mL^-1^) were grown in YPD plus 0.6 M NaCl, harvested, washed and suspended in different incubation media. Bar code is the same as in Fig 5A. Incubation media were composed of 1% yeast extract and 2% peptone without a carbon source (YP). YP base was supplemented with sorbitol (1.2 M) NaCl (0.6 M) or KCl (0.6M). Respiration was induced by the addition of 25 mM glucose. Additions: 3 mM SHAM, 0.5 mM potassium cyanide. * *p* < 0.05 respect to Aox (dark bars) or cytochrome-dependent (light bars)respiration.

The data presented above show that Aox functions as an escape-valve for electrons under circumstances of overflow in the cytochrome pathway for stationary phase cultures. To test this, cells grown in YPD-media without sodium were harvested and shifted immediately to YPD- media with/without osmolites and respiration was analyzed. This protocol eliminates the effects of osmolites on Aox gene expression seen in sodium/potassium/sorbitol-containing cultures since expression is only observed after 30 minutes of incubation. Fig 5A shows that cells shifted to the same media (white bars) presented almost the same respiratory activity as the mother culture. However, when a second aliquot of this culture was incubated in osmolite-containing media, Aox-dependent respiration (dark gray bars) increased at the expense of the cytochrome pathway (light gray bars), indicating that Cox-dependent respiration was partially compromised when cells are shifted to hyperosmotic conditions.

In a parallel experiment, cells growing in sodium media (where Aox expression is higher) were treated in the same way as panel A and respiration quantified. Fig 5B shows that independently of the expression level of Aox, the extracellular hyperosmotic environment affects the cytochrome pathway (light gay bars), which relies on Aox to sustain an adaptive mechanism under hyperosmotic conditions (dark gray bars). It is important to note that the protein levels of CoxIII and Aox remain constant during the 15 minutes of the experiment (not shown).

**Fig 6:**
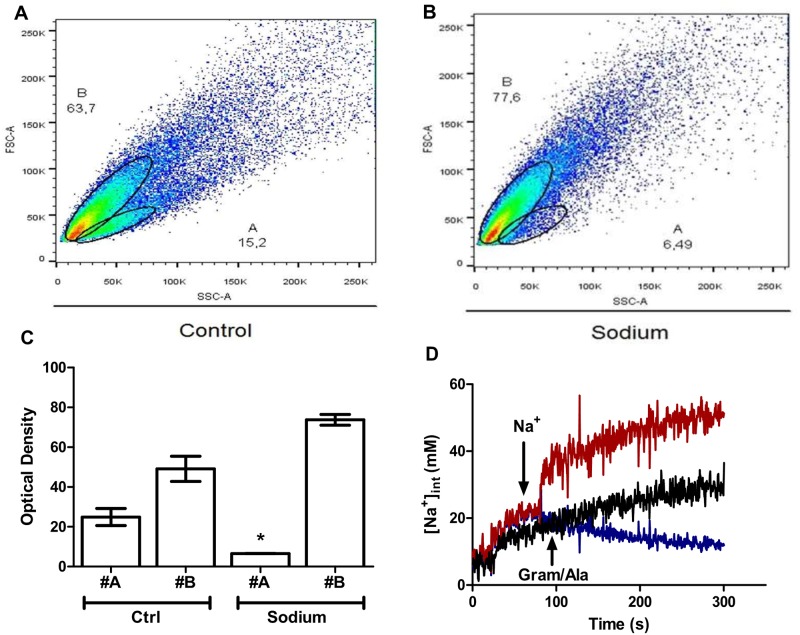
Sodium is incorporated into cells without loss of cell volume. (A-B) Cells grown in YPD media until logarithmical phase were washed and suspended in buffer (20 mM Hepes pH 6.8) (A) or buffer supplemented with 0.6 M NaCl (B) and cells analyzed by flow cytometry. Letters A and B inside these figures represent the two subpopulations observed by flow cytometry. (C) Percentage of cells in each subpopulation for each treatment. (D Cellular sodium uptake under the same conditions used in the flow cytometry assays. Representatives traces. Black trace, control (cells in 20 mM Hepes pH 6.8). Red, cells in buffer with subsequent addition of 0.6 M NaCl. Blue trace, as in black trace but where indicated 10μM gramicidin plus 10 μM alamethicin was added to the incubation media. Glucose (25 mM) was included in all the experiments to energize the cells. * p < 0.05 respect to control (YPD) samples.

Since an osmotic effect seems to be involved, cellular size would be expected to be affected because of the outward movement of water when cells are shifted to sodium media. When looking for any change in cell structure because of sodium incubation, cell size was found to be unaffected by the presence of 0.6 M sodium media. However, complexity (or granularity) increased under these conditions, indicating that internal osmolarity is finely regulated in this yeast (Fig 6A–6C). It has been demonstrated that *D*. *hansenii* actively transports sodium and store it in large vacuoles [17]. In this regard, the greater complexity observed in the cytometry analysis is explained by the increase in the size of vacuoles, differently from *S*. *cerevisiae*, where vacuole fragmentation is observed after a salt insult [18]. To corroborate that under our experimental conditions the increase in complexity is the result of sodium internalization, we measured sodium uptake with the fluorescent probe SBFI-AM. As observed in Fig 6D, when cells are exposed to 0.6 M sodium chloride, this salt is avidly internalized by *D*. *hansenii* in an energy-dependent process, supporting the idea that this yeast is sodium-including [19].

### Osmolarity affects viability and the stress response

**Fig 7:**
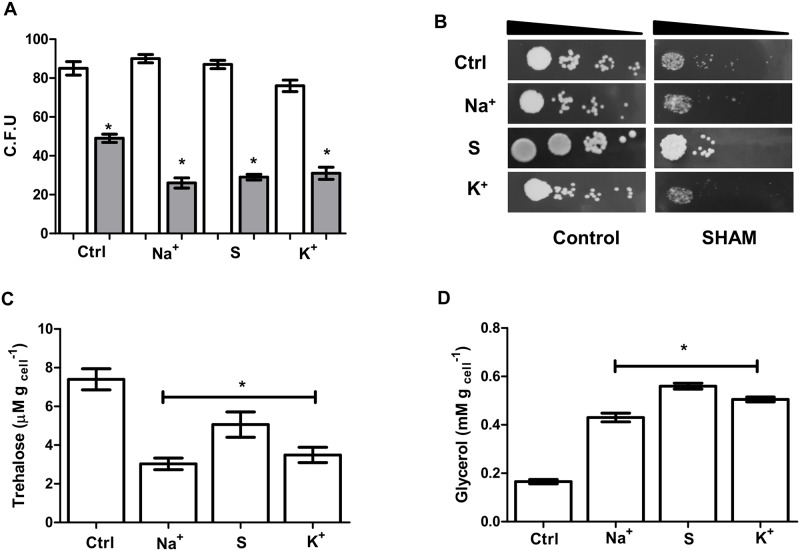
Alternative oxidase activity is necessary for growth in high osmolarity media. (A) Cells were grown in YPD media with and without NaCl, KCl or sorbitol (white bars). Some plates were supplemented with 3 mM SHAM (gray bars) to determine the effect of Aox on hyperosmotic media. Samples were spread on YPD plates at 28°C and colonies were counted after 2 days. (B) Representative plates of serially diluted samples to show the effect of 3 mM SHAM on cell grown in the presence of the indicated osmolites. Ctrl, YPD media, Na^+^, YPD plus 0.6 M NaCl, S, YPD, YPD media plus 1.2 M sorbitol, K^+^, YPD media plus 0.6 M KCl. (C-D) Compatible solute production in the cells grown in the presence of the indicated osmolites. Cells grown in the indicated media were lysed and extracts assayed for trehalose (C) and glycerol (D) content. * *p* < 0.05 respect to control cells.

Next, we determined if higher Aox expression affects the viability of this yeast. In Fig 7A, we show that cells diluted and spotted on plates containing the Aox inhibitor, SHAM, presented reduced growth, suggesting that proper Aox activity is necessary for survival (Fig 7A and 7B) in all tested condition although a more stringent phenotype is observed under hyperosmotic conditions where mitochondrial respiration is more dependent on Aox activity (Fig 7A, gray bars).

Yeasts actively produce compatible solutes in order to sustain viability under stationary phase or latency conditions. When two of these substances were quantified (glycerol and trehalose), only glycerol was accumulated in sodium, potassium or sorbitol-containing media (Fig 7C and 7D), confirming data from other groups [20, 21] and reinforcing the role of mitochondria and Aox in the cellular response to saline environments.

**Fig 8:**
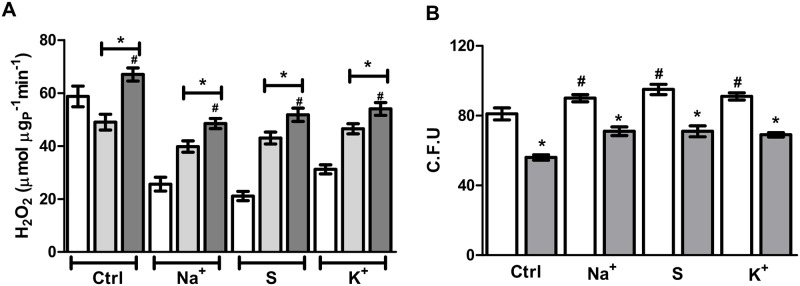
High osmolarity promotes higher resistance to oxidative stress. (A) Hydrogen peroxide production rates in isolated mitochondria. Isolated mitochondria (0.5 mg/mL) from cells in each experimental condition were assayed for hydrogen peroxide production using 1 mM NADH as a respiratory substrate. White bars, basal hydrogen peroxide production. Light gray bars, hydrogen peroxide production in the presence of 100 μM SHAM. Dark gray, hydrogen peroxide production in the presence of 1 μM antimycin A. (B) Cells grown YPD media (white bars) or YPD+SHAM (3 mM, gray bars) were treated with 10 mM hydrogen peroxide for 10 minutes, samples were plated on YPD and incubated at 29°C for 2 days. Colonies were manually counted. *p < 0.05 respect to basal oxidant production (white bars, panel A), #*p* < 0.05 respect to oxidant production in the presence of SHAM (Light gray bars, panel A) For panel B, *p < 0.05 respect to control cells with SHAM. # *p* < 0.05 respect to control without SHAM.

Finally, since reactive oxygen species (ROS) are produced as a side-reaction during electron transfer, we asked if Aox would have a role on the production of these species. To test this, hydrogen peroxide (H_2_O_2_) production was quantified under the same growth conditions. Fig 8A shows that higher Aox activity decreased the basal production of hydrogen peroxide by preventing electron leak from the ubiquinol pool. When SHAM or antimycin (light and dark gray bars respectively) were included in the incubation media, an increase in the production of mitochondrial oxidants was observed. This result corroborates that effectively Aox avoids electron overflow (and leak) through the respiratory chain. Moreover, when cells were temporarily exposed to H_2_O_2_ and the viability assessed, a higher number of colonies was resistant to this oxidant, which correlates with the Aox expression level. (Fig 8B). Moreover, when cells were previously incubated in the presence of SHAM and treated with hydrogen peroxide as in control cells a decreased in viability was observed independently of the osmoticum present. Overall, these results suggest that Aox is involved in the homeostasis of mitochondrial oxidants, which is reflected in the increased resistance toward exogenous oxidants.

## Discussion

In this report, we describe the effects of high osmolarity on respiratory activity of the halotolerant yeast *D*. *hansenii*, demonstrating how an alternative enzyme of the respiratory chain is involved in this adaptive mechanism. Despite the well-known function of this protein under abiotic stress, little is known about the function of Aox under permissive conditions for this yeast (i.e. high osmolarity).

Here, we observed that instead of acting as an electron sink and energy-dissipating protein, Aox in *D*. *hansenii* allows their mitochondria to maintain a stable membrane potential when the cytochrome pathway is compromised. This has profound relevance in the context of the adaptive response to hyperosmotic environments, since this means that *D*. *hansenii* is able to support active recycling of skeleton carbons by the Krebs cycle in addition to producing a small but significant quantity of ATP generated by the protonmotive force maintained upon functional coupling between complex I and Aox. Indeed, when a cell is suddenly transferred from low to high salinity or vice versa, Aox responds by maintaining a continuous electron flux through the respiratory chain, confirming its importance in halotolerance.

Evidence presented in this report supports the view that *D*. *hansenii* is a sodium-including yeast [22], since no changes were observed in cell size in sodium media. Other yeast, such as *S*. *cerevisiae*, transiently decrease their internal volume as a thermodynamic response to osmotic pressure [23], while *D*. *hansenii* internalizes sodium as a response to osmotic changes instead of decreasing its cell size. In this regard, vacuoles, by means of their H^+^ V- ATPase and the Na/H^+^ exchangers, support the high sodium storing capacity of this yeast [24]. Importantly, mitochondria play a key role in maintaining a high energy levels to support the activity of these ATPases. As stated above, not only the canonical respiratory chain but also the alternative pathway described here support the production of mitochondrial ATP. Indeed, we find that decreasing Aox activity inhibits cell survival in the presence of high external osmolite concentrations.

Overall, our results point to Aox as a regulator of mitochondrial metabolism under hyperosmotic conditions, that acts by maintaining redox state, energy availability and electron flow in the respiratory chain. This report improves our understanding of the protective role played by Aox in plants, fungi and some protozoa against the most diverse experimental stresses [25–26].
